# *Premna grandipaniculata* (Lamiaceae, Premnoideae), a remarkable new species from north Myanmar

**DOI:** 10.3897/phytokeys.94.22033

**Published:** 2018-01-29

**Authors:** Yun-Hong Tan, De-Rong Li, Shi-Shun Zhou, Yong-Jun Chen, Gemma L.C. Bramley, Bo Li

**Affiliations:** 1 Southeast Asia Biodiversity Research Institute, Chinese Academy of Sciences, Yezin, Nay Pyi Taw 05282, Myanmar; 2 Centre for Integrative Conservation, Xishuangbanna Tropical Botanical Garden, Chinese Academy of Sciences, Mengla, Yunnan 666303, China; 3 College of Agronomy, Jiangxi Agricultural University, Nanchang, Jiangxi 330045, China; 4 Herbarium, Royal Botanic Gardens Kew, Richmond, Surrey TW9 3AE, UK

**Keywords:** Morphology, paniculiform inflorescence, *Premna*, spike-like thyrses

## Abstract

A remarkable new *Premna* species from Myanmar, *P. grandipaniculata* Y.H.Tan & Bo Li (Lamiaceae), is here described and illustrated. It differs from all known congeneric taxa by having huge complicated panicles which have tertiary branches formed by spike-like thyrses. In *Premna*, such a spike-like thyrse is found in *P. bracteata* and *P. interrupta*, but those species can be easily distinguished from *P. grandipaniculata* by their habit, indumentum, leaf size and inflorescence structure.

## Introduction

**Figure 1.**
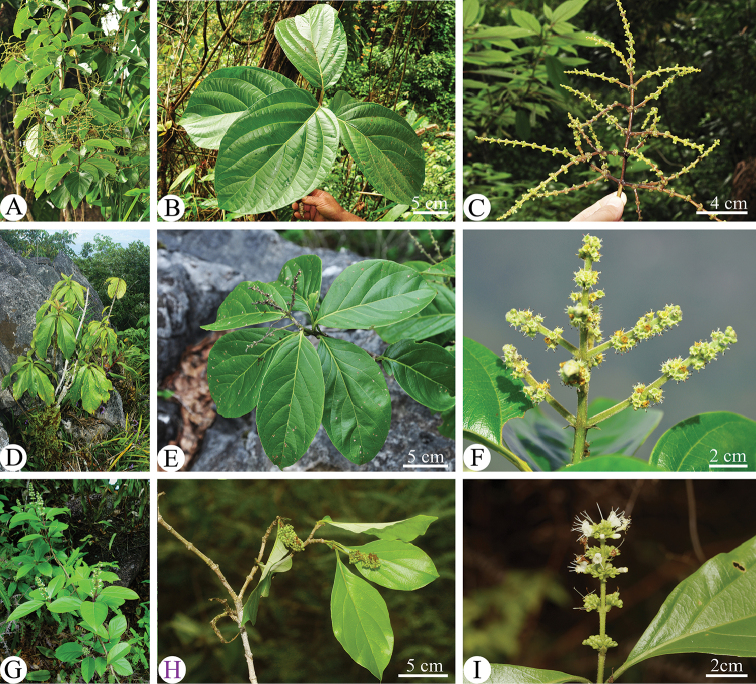
Morphological comparisons amongst *Premna grandipaniculata* (**A–C**), *P. bracteata* (**D–F**) and *P. interrupta* (**G–I**). **A, D, G** habit **B, E, H** branchlets with leaves **C, F, I** inflorescences.

Myanmar is an important component of the Indo-Burma biodiversity hot-spot (Conservation International, available at: http://www.biodiversityhotspots.org/xp/Hotspots/indo_burma/) and its northern region emerges in the Himalaya Centre, one of the globally richest plant diversity centres (Rafiqpoor et al. 2005, Brooks et al. 2006). However, for nearly half a century, there has been a great shortage of critical floristic surveys in northern Myanmar. A recent surge of field explorations in this region coordinated by the Southeast Asia Biodiversity Research Institute, Chinese Academy of Sciences, has resulted in the discovery and description of new species (e.g. Aung et al. 2017, Liu et al. 2017, Tan et al. 2017, Yang et al. 2017a, b, c). During fieldwork in 2016 and 2017, the first author encountered and collected an unusual *Premna* L. plant, a huge woody climber bearing large, complicated paniculiform inflorescences and large, suborbicular, glabrous leaves. The terminal branches of the inflorescence are spike-like thyrses formed by sessile cymes laxly arranged on the axis. After checking and comparing the plant with all known congeneric taxa, it was found that such an inflorescence is similar to that of *P. bracteata* Wall. ex C.B. Clarke and *P. interrupta* Wall. ex Schauer, but the plant differs significantly from those two species in many aspects (Figure 1, Table 1). Thus, it was confirmed that it represents a remarkable undescribed new species and it is presented here.

**Table 1.** Morphological comparisons amongst *Premna grandipaniculata*, *P. bracteata* and *P. interrupta*.

Taxonomic traits	*P. grandipaniculata*	*P. bracteata*	*P. interrupta*
**Habitat**	large climbers	small trees	erect to climbing shrubs
**Indumentum**	nearly glabrous throughout	branchlets, petioles, leaf veins and inflorescences densely pubescent	branchlets, petioles, leaf veins and inflorescences densely pubescent
**Leaves (length** × **width)**	14–23 × 10.5–17.5 cm, leathery, broadly ovate to subrounded, base subrounded, rounded to slightly cordate, apex acute	6.5–17 × 5.5–10 cm, subleathery, oblong to ovate, base broadly cuneate to subrounded, apex abruptly acuminate or rarely obtuse	6.0–12 × 4.0–7.5 cm, papery to subleathery, rhomboid-elliptic, ovate-oblong to obovate, base acuminate, cuneate to broadly cuneate, apex acuminate, acute or rarely obtuse
**Inflorescence**	a large complicated panicle with 4–6 pairs of secondary branches and 2–3 pairs of tertiary branches, terminal branches are spike-like thyrses with sessile cymes	a panicle with 2–4 pairs secondary branches, each is a spike-like thyrse with sessile cymes	a single spike-like thyrse formed by sessile cymes

## Methods

Both herbarium specimens and living branches of the putative new species and of *P. bracteata* and *P. interrupta* were observed under a stereo dissecting microscope (StereoZoom Leica S8 APO, Leica Microsystems 2017) and measured using a ruler and a micrometer. High resolution images of the type specimens of *P. bracteata* (held at M and K, acronyms according to Thiers 2017) and of *P. interrupta* (held at BM, E, K, and P) were consulted on JSTOR Global Plants (http://about.jstor.org/, accessed 15 August 2017). Type specimens held at K, (barcode no. K001114139 for *P. bracteata*, K000884639, K000884640 and K001114151 for *P. interrupta*) were examined in the Herbarium. The conservation status of the new species was evaluated based on the guidelines of the International Union for Conservation of Nature (IUCN 2012).

## Taxonomy

### Premna grandipaniculata

Taxon classificationPlantaeLamialesLamiaceae

Y.H.Tan & Bo Li sp. nov.

urn:lsid:ipni.org:names:77175495-1

Figures 1A–C , 2

#### Diagnosis.

This species is distinguishable by its huge complicated paniculiform inflorescences. *Premna grandipaniculata* shares the same primary inflorescence structure with *P. bracteata* and *P. interrupta*, but is distinct from the latter two in its spike-like thyrses forming a panicle with tertiary branches (vs. with secondary branches in *P. bracteata*, while without branches in *P. interrupta*) and in having nearly glabrous branchlets, petioles, leaves and inflorescences (vs. densely pubescent throughout in the latter two species) (Table 1).

#### Type.

MYANMAR. Kachin State, Putao District, ca. 2–3 miles from Wasandum village, 27°29'00.29"N, 97°12'01.48"E, Alt. 1050 m, 29 April 2016, *Y.H. Tan & S.S. Zhou 20160031* (holotype: HITBC!; isotypes: HITBC!,RAF!, JXAU!).

#### Description.

*Woody* climbers. *Branches* grey, terete, robust, nutant, without an interpetiolar ridge. *Branchlets* purplish brown, with densely small white elliptic lenticel stomentose, without bracts at the base. *Leaves* simple, opposite-decussate, glabrous, broadly ovate to suborbicular, leathery, 14–23 × 10.5–17.5 cm, apex acute, base subrounded, rounded to slightly cordate, margin entire; veins 4–7 pairs, abaxially raised and adaxially slightly compressed, secondary veins curved and jointed near margin; petiole 2.4–3.5 cm long, purplish dark green, slightly inflated, purplish furrowed on upper part. *Inflorescences* terminal, a large complicated panicle with tertiary branches, 18–30 cm long, 12–20 cm wide, peduncle nearly glabrous, terminal branches spike-like thyrses, 10–20 cm long, formed by sessile cymes laxly arranged on axis; bracts ovate, 3.0–10 mm long, easily deciduous; bracteoles subulate, tiny. *Calyx* campanulate, 2.0–2.5 mm long, outside minutely brownish pubescent, 2-lipped; lips entire, or upper lip emarginate and ciliate, apex subrounded. *Corolla* green to greenish white, subglabrous, outside glandular, inside densely white villose around throat, 4-lobed; lobes broadly obovate, apex subrounded. *Stamens* 4, equal, exserted; anther purple. *Ovary* oblong, 1.0–1.5 mm long, glabrous, glandular; style white, slender, 3.5–4.5 mm long. *Fruits* drupaceous, narrowly obovate, 4.0–5.0 × 2.5–3.5 mm, yellowish brown.

**Figure 2.**
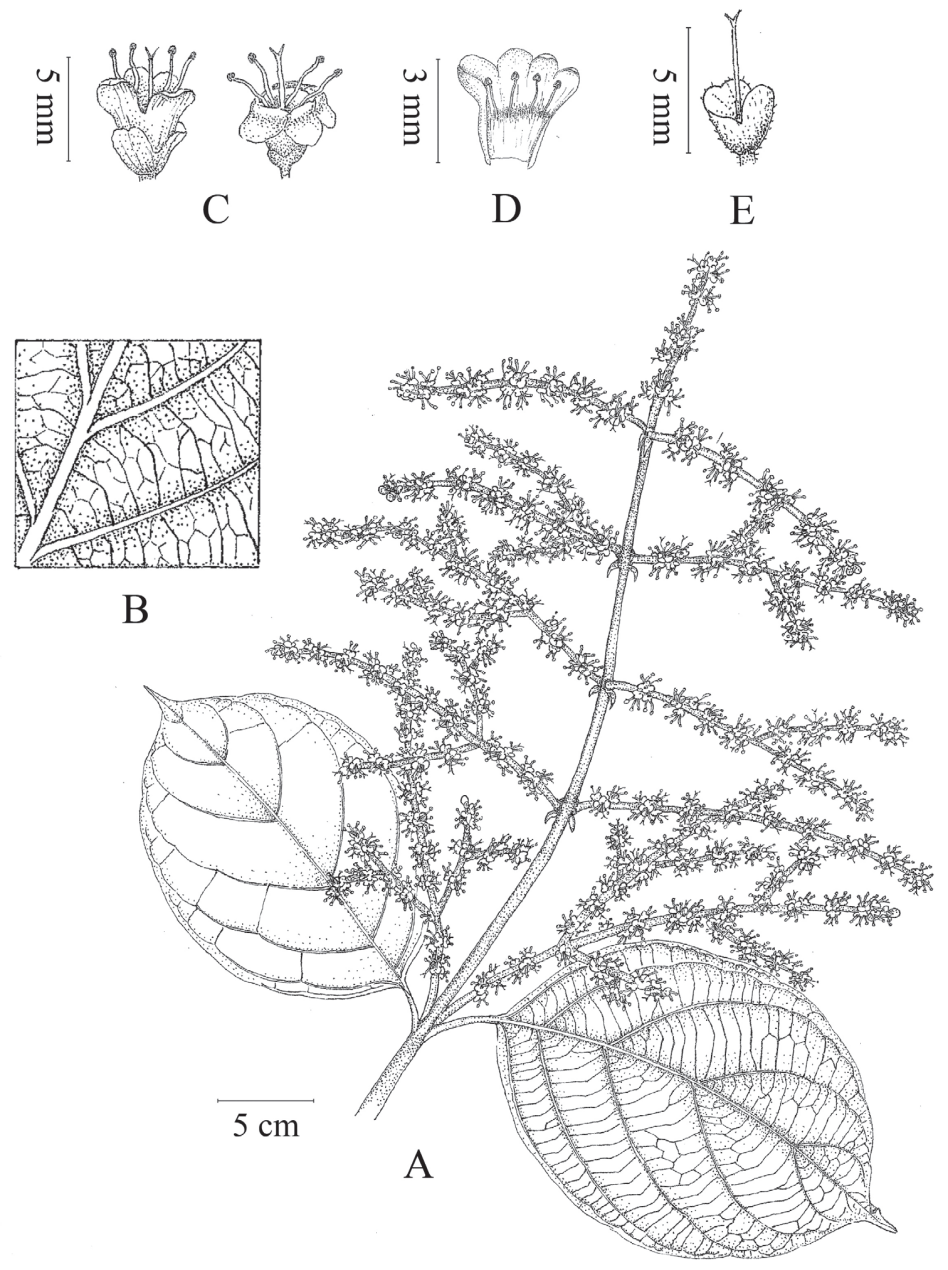
Line drawing of *Premna grandipaniculata* Y. H. Tan & Bo Li, sp. nov. **A** a branchlet with leaves and inflorescence **B** abaxial surface of leaf blade **C** flowers **D.** dissected corolla and stamens in a bud **E** calyx and style.

#### Phenology.

Flowering was observed from early March to April and fruiting from late May to late June.

#### Distribution.

The species is currently known only from the type locality of Putao, Kachin State, northern Myanmar, grows in tropical montane forests, at an elevation 700–1200 m a.s.l.

#### Etymology.

The specific epithet “*grandipaniculata*” indicates that the species bear large complicated paniculiform inflorescences.

#### Preliminary conservation status.

This species is only known from a single locality in Myanmar and as the habitat, in which it is found, is threatened by deforestation (author’s personal observation), it is categorised as critically endangered under criteria B and D following IUCN Red List Categories (IUCN 2012).

#### Note.

**Figure 3.**
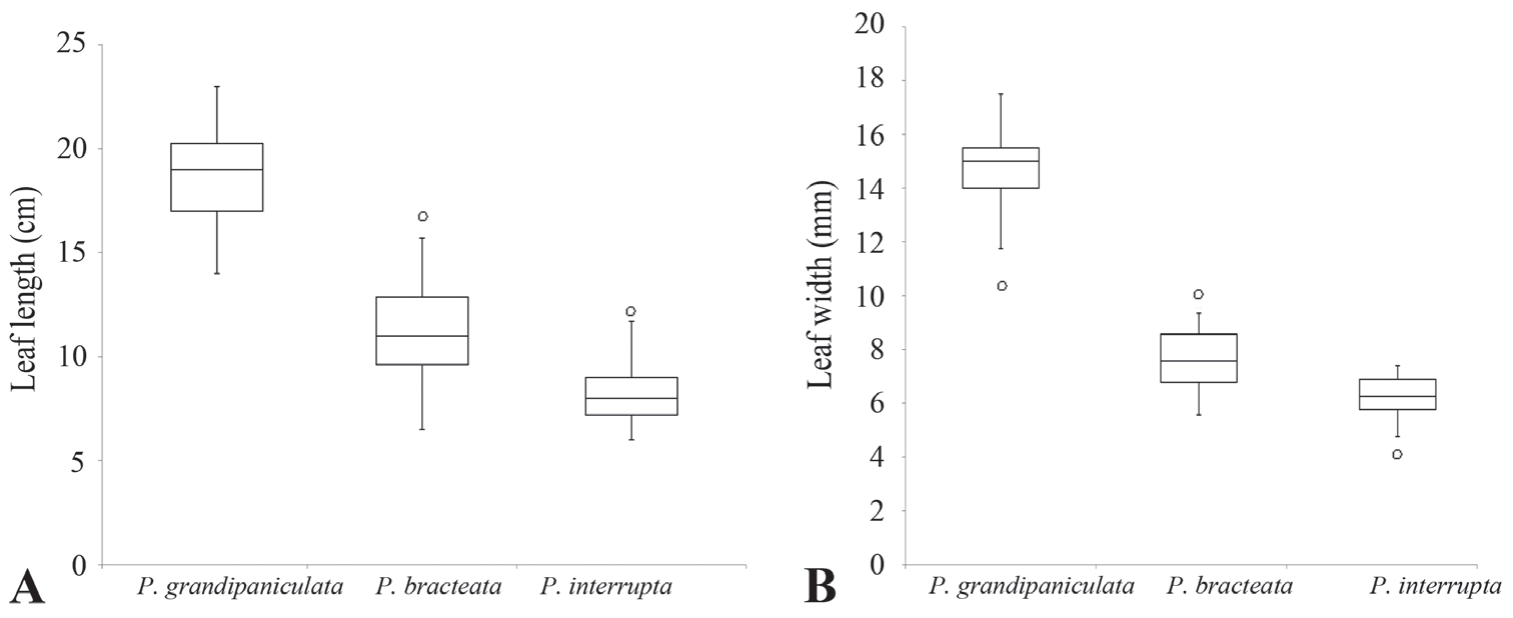
Box plots of two quantitative characters, leaf length (**A**) and leaf width (**B**), of *Premna grandipaniculata*, *P. bracteata* and *P. interrupta*. The boxes (rectangle region) represent the interquartile range and the whiskers (vertical line) represent the range excluding the outliers (circles). The three upper, middle and lower lines on the boxes represent the 75%, 50% and 25% of the variables, respectively. The upper and lower ends of the whiskers represent the maximum and minimum values of the variables, respectively. The circles represent the single value, where the variable value exceeds 1.5 times the difference between the 75% and 25%.

The most noticeable trait of the new species is its huge complicated paniculiform inflorescence. After examination, it was found to be formed by tertiary branches of spike-like thyrses. Such a spike-like thyrse is a rare type of inflorescence in *Premna*, currently found in only two species, *P. bracteata* and *P. interrupta*. In *P. interrupta*, sessile cymes form a single spike-like thyrse without branches, while in *P. bracteata* the lower parts of inflorescence bear 2–4 pairs of secondary branches. Besides its inflorescence structure, *P. grandipaniculata* also differs from *P. bracteata* and *P. interrupta* in having larger leaves (Figure 3), nearly glabrous branchlets, petioles and inflorescences (Table 1).

Geographically, *P. bracteata* is mainly recorded from the southern and eastern slopes of the Himalayas with several collections from Bangladesh, Bhutan, Myanmar, north eastern India and southeast Tibet of China and has also been collected from a rare and isolated population in Xishuangbanna, south Yunnan of China (Chen and Gilbert 1994, Govaerts et al. 2008, unpublished data). *Premna interrupta* frequently occurs from southwest China (Guangxi, Guizhou, Sichuan, Xizang and Yunnan provinces) to southern and south east Asia (Bangladesh, Bhutan, Cambodia, Laos, Myanmar, north eastern India, Nepal, Peninsular Malaysia, Thailand and Vietnam) (Chen and Gilbert 1994, Govaerts et al. 2008). In Myanmar, *P. bracteata* is recorded from Chin, Mandalay and Sagaing, while *P. interrupta* from Kachin, Rakhine and Sagaing (Kress et al. 2003). In Kachin States, the distribution of *P. interrupta* overlaps that of *P. grandipaniculata* and their habitat and habit are also similar, but *P. grandipaniculata* significantly differs from *P. interrupta* in morphology as mentioned above.

## Supplementary Material

XML Treatment for Premna grandipaniculata
